# HMMvar-func: a new method for predicting the functional outcome of genetic variants

**DOI:** 10.1186/s12859-015-0781-z

**Published:** 2015-10-30

**Authors:** Mingming Liu, Layne T. Watson, Liqing Zhang

**Affiliations:** 1 Department of Computer Science, Virginia Polytechnic Institute & State University, Blacksburg, USA; 2 Department of Mathematics, Virginia Polytechnic Institute & State University, Blacksburg, USA; 3 Department of Aerospace and Ocean Engineering, Virginia Polytechnic Institute & State University, Blacksburg, USA

**Keywords:** Genetic variants, Functional outcome, Hidden Markov model

## Abstract

**Background:**

Numerous tools have been developed to predict the fitness effects (i.e., neutral, deleterious, or beneficial) of genetic variants on corresponding proteins. However, prediction in terms of whether a variant causes the variant bearing protein to lose the original function or gain new function is also needed for better understanding of how the variant contributes to disease/cancer. To address this problem, the present work introduces and computationally defines four types of functional outcome of a variant: gain, loss, switch, and conservation of function. The deployment of multiple hidden Markov models is proposed to computationally classify mutations by the four functional impact types.

**Results:**

The functional outcome is predicted for over a hundred thyroid stimulating hormone receptor (TSHR) mutations, as well as cancer related mutations in oncogenes or tumor suppressor genes. The results show that the proposed computational method is effective in fine grained prediction of the functional outcome of a mutation, and can be used to help elucidate the molecular mechanism of disease/cancer causing mutations. The program is freely available at http://bioinformatics.cs.vt.edu/zhanglab/HMMvar/download.php.

**Conclusion:**

This work is the first to computationally define and predict functional impact of mutations, loss, switch, gain, or conservation of function. These fine grained predictions can be especially useful for identifying mutations that cause or are linked to cancer.

**Electronic supplementary material:**

The online version of this article (doi:10.1186/s12859-015-0781-z) contains supplementary material, which is available to authorized users.

## Background

Mutations contribute to human evolution and disease development. Over 79 million genetic variants have been identified in 2535 humans from 26 populations around the world (the 1000 Genomes project, 06/2014). The sheer enormity of the number of these variants poses a grave challenge for researchers to empirically examine their individual or collective phenotypic or pathological effects and identify the ones that are important determinators for phenotypes or diseases. Consequently, to help narrow down target variants that may have phenotypic and/or pathological effect, various computational tools (e.g., [1–6]) have been introduced to predict the effect of genetic variants. Specifically, these tools provide either a quantitative score indicating the degree of deleteriousness of the variant (e.g., [1–4]), or a qualitative statement of whether the variant is deleterious or neutral (e.g., [7]).

**Fig. 1:**
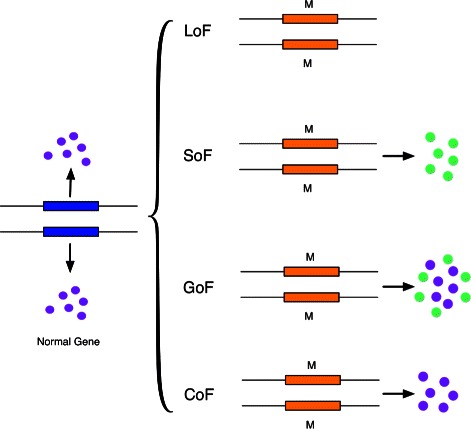
The consequences of loss, switch, gain or conservation of function mutations (M). The normal gene is indicated by a blue box and the mutated gene by an orange box. The original functions are represented by blue circles and the new functions by green circles

However, none of the existing tools can provide fine grained prediction on the likely cellular outcome of mutations, such as gain, loss, switch, or conservation of function. Biologically, loss of function (LoF) mutations cause the gene product to have reduced activity or complete loss of function; gain of function (GoF) mutations change the gene product to have a new and possibly abnormal function; switch of function (SoF) mutations cause the gene product to switch from one set of functions to another set of functions [8], and thus may involve both loss of the original functions and gain of new functions; conservation of function (CoF) mutations, coined in this study, refer to mutations that are neutral and do not alter gene functions. Figure 1 illustrates these definitions.

Fine grained prediction of the effect of mutations on function has important applications in disease and cancer research. For instance, two important classes of genes, oncogenes and tumor suppressor genes, when mutated, can both lead to cancer. However, the effects that mutations have on these cancer causing genes are almost the opposite. Mutations in oncogenes can keep the genes stuck in a state of constant or increased activity. A proto-oncogene converted into an oncogene generally involves gain of function. For example, in the proto-oncogene BRAF, there is a well-known gain of function mutation, V600E, that replaces the amino acid valine (V) with the amino acid glutamic acid (E) at position 600. The V600E mutation enables a 500-fold increased activation in BRAF, stimulating the constant activation of the mitogen-activated protein kinase (MEK) signaling that leads to a tumor cell [9]. This mutation has been found frequently in the skin cancer melanoma [10]. Contrarily, mutations in a tumor suppressor gene cause the gene to lose the ability to prevent or “suppress” abnormal cells from developing into full-blown tumors, and therefore are essentially loss of function mutations. An example can be seen in PTEN, one of the most commonly down-regulated tumor suppressor genes in cancer genomes. Substitutions for some of its important residues, such as D92 and H93, result in significantly reduced PTEN function [11]. Therefore, identifying different types of mutations in terms of functional impacts helps understand the driven event and the identification of novel targets, which is crucial for the development of targeted disease and cancer therapeutics.

Earlier work addresses prediction of the functional type of variants [12, 13] by trying to identify activating variants, but none provides a precise computational definition for all these classification types: loss, gain, switch, and conservation of function. This work computationally classifies genomic variants into four types on the basis of previous work on functional effect prediction of genetic variants using HMMvar [14], a method based on the principle of evolutionary conservation and hidden Markov models (HMMs). Multiple sequence alignment (MSA) captures the evolutionary information within homology sequences. Evolutionary analysis provides a powerful tool for predicting the functional impact of mutations. Presumptively, a profile HMM built from the MSA is an implicit representative of a set of functions of the protein family. From each protein subfamily cluster, a HMM is built and used to score the variants. Based on the “fitness” of a sequence within a family or across subfamilies, different types of mutations are defined. The loss of function mutations weaken the fitness of the mutant type sequence with the protein family, whereas the gain of function mutations make the mutant type sequence fit better than the wild type sequence in one of the subfamilies. The switch of function mutation is a combination of loss of function and gain of function, which causes the mutant type sequence to lose functions from the original protein family but gain functions from other subfamilies. Conservation of function means the mutation does not cause any functional changes (see the Methods section for details).

## Methods

### Data Sources

111 thyroid stimulating hormone receptor (TSHR) mutations (Additional file 1: Table S1) are extracted from the TSH Receptor Mutation Database II [15]. They are all nonsynonymous single nucleotide polymorphisms (SNPs). 61 out of 111 are gain of function that constitutionally activate the receptor independently of TSH; the remaining 50 are loss of function that result in the loss of TSH sensitivity.

Mutations on tumor protein p53 (TP53), a set of 2,565 SNP mutants (Additional file 1: Table S2), and corresponding biological activity levels were obtained from the database IARC TP53 [10]. The mutants were partitioned into four classes in terms of transactivity level: nonfunctional, partially functional, functional (wildtype), and supertrans (higher activity than wildtype) [11]. Transactivity level was measured by eight promoter-specific activity levels and the classification was made in terms of the median of these eight levels. Mutations are classified as “nonfunctional” if the median is <20, “partially functional” if the median is >20 and <75, “functional” if the median is >75 and <140, and “supertrans” if the median is >140.

For the epidermal growth factor receptor (EGFR) gene and the proto-oncogene B-Raf (BRAF) gene, 124 activating mutations that are targeted by selective inhibitors to inhibit only mutated genes [16] are evaluated (Additional file 1: Table S3–S4).

To validate HMMvar-func’s ability in predicting switch of function, the four mutations in RAC1, PTPRD, MAP2K4, and CDH1, identified by [8] to be likely “switch of function” mutations, are examined.

### Build multiple HMMvars

HMMvar [14] quantitatively predicts the functional effects of variants. It builds a HMM based on the MSA of a set of homologous sequences to the wild type sequence. Then the wild type protein sequence and mutant type protein sequence are matched against the HMM, respectively. HMMvar provides a score to measure the fitness or similarity between the sequence and the “protein family” represented by the HMM. If the mutant type sequence fits almost the same as the wild type sequence, the mutation has little effect on the protein function. To identify different types of mutations, a MSA of homologous sequences is clustered and each cluster is viewed as a “subfamily”, which captures specific functions. If a mutant sequence fits better than the corresponding wild type sequence in one of the “subfamilies”, then probably the variant enables the protein to “acquire” new functions. With this assumption, clustering the homologous sequences, including the query sequence, identifies “subfamilies”, each of which represents a functional profile. The detailed steps are given below.

**Fig. 2:**
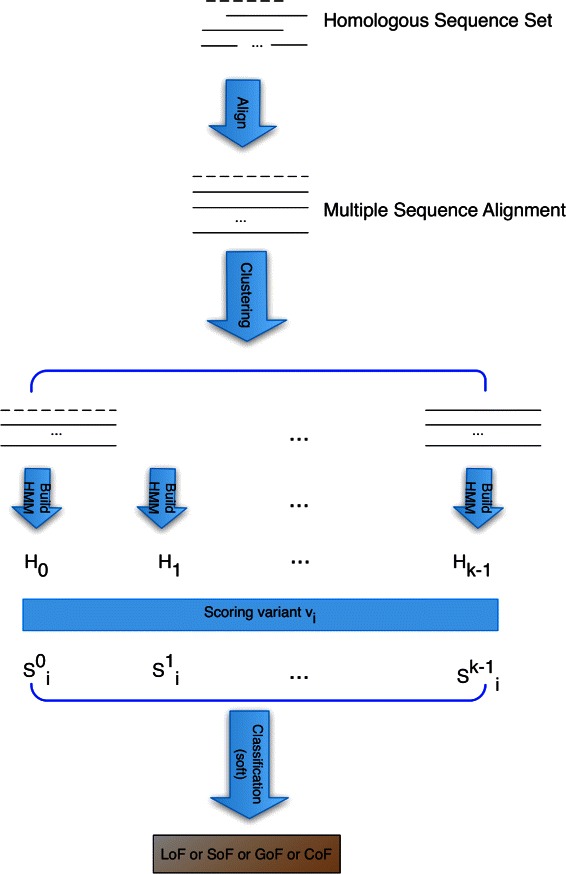
Flowchart of the classification procedure. The dashed line represents the wild type sequence

The pipeline is shown in Fig. 2. First, homologous sequences to the wild type protein are identified by PSI-BLAST [17] against the UniProt90 [18] database. Then the homologous sequences are aligned by the multiple sequence alignment algorithm MUSCLE [19] with parameters “-maxiters 1 -diags -sv -distance1 kbit20_3”. The number of iterations (=1) is specified by the “-maxiters” option. The “-diags” option enables an optimization for speed. The “-distance1” option specifies the distance measure. These options enable Muscle to run the fastest possible. To ensure the quality of the MSA, further processing was performed. First, redundant sequences are removed. If the identity percentage between the aligned positions of any two sequences in the alignment exceeds a threshold (95 %), the shorter sequence is discarded. Then low quality columns (those with the number of gaps exceeding a threshold (99 %)) are discarded. Given a variant, a region of the MSA is selected by left and right extension from the position of the variant, keeping the query sequence consecutive in the MSA. If the length of the selected region of the MSA is less than 10 base pairs, more extensions are continuously performed considering the quality of the columns (e.g. the percentage of gaps is less than 10 %). Finally, empty rows are removed (rows with all gaps).

With the postprocessed MSA, the combinatorial entropy optimization (CEO) algorithm [20] is used to perform the clustering. This algorithm minimizes the sum of the difference between observed and expected entropy across different clusters over all the positions in the MSA. Minimizing the combinatorial entropy yields an optimized partition of the MSA such that the columns are conserved in a subfamily (cluster) but differ between subfamilies. For each of the clusters, a profile HMM is built, which represents a “subfamily” of specific functions that differ from those of the target cluster; then HMMvar can be used to score the variants. Denote these “subfamilies” by *C*_0_, *C*_1_, …, *C*_*k*−1_, where *C*_0_ is the target cluster that contains the wild type sequence, and the corresponding HMMs as *H*_0_, *H*_1_, …, *H*_*k*−1_. Only the clusters with size greater than one are used for prediction in the pipeline.

### Classification of mutations

**Fig. 3:**
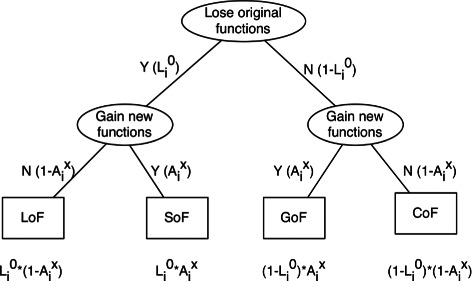
The probability combination rule to calculate the confidence score for the classification of mutations

The HMM in HMMvar [14] is used to predict the degree of harm in the variants and only one HMM is built from the MSA of all the homologous sequences. In this paper, multiple HMMs are built for prediction, one HMM for each of the *k* clusters. For a given variant *v*_*i*_, let ${S_{i}^{m}}$ (0≤*m*≤*k*−1) denote the quantitative HMMvar score of variant *v*_*i*_ obtained from *H*_*m*_. Note that *H*_0_ is the HMM built from the target group *C*_0_ where the wild type sequence clustered (the dashed line shown in Fig. 2), thus ${S_{i}^{0}}$ is the score of variant *v*_*i*_ calculated from *H*_0_. Since the scores are sensitive to the clustering, a soft classification is used. Given a variant *v*_*i*_, the probability ${L_{i}^{0}}$ of losing the original functions from *C*_0_ and the probability ${A_{i}^{x}}$ of acquiring new functions from *C*_*x*_ are defined by

$$\begin{array}{@{}rcl@{}} {L_{i}^{0}} & = & \frac{1}{1+e^{-({S_{i}^{0}}-t)}},\\ {A_{i}^{x}} & = & \frac{1}{1+e^{-(t-{S_{i}^{x}})}}, \end{array} $$

where ${S_{i}^{0}}$ is the score calculated from *H*_0_, ${S_{i}^{x}} = \min \limits _{1\le j \le k-1} {S_{i}^{j}} $, and *t* is the user defined cutoff. The logistic functions correspond to assuming that the logarithms of the odds ratios for ${L_{i}^{0}}$ and ${A_{i}^{x}}$ are linear in the threshold *t*. Then from combinatorial probability, the confidence scores are ${L_{i}^{0}}*(1-{A_{i}^{x}})$, ${L_{i}^{0}}*{A_{i}^{x}}$, $(1-{L_{i}^{0}})*{A_{i}^{x}}$, and $(1-{L_{i}^{0}})*(1-{A_{i}^{x}})$ for loss of function (LoF), switch of function (SoF), gain of function (GoF), and conservation of function (CoF), respectively. The binary tree in Fig. 3 demonstrates how the confidence score for different types is calculated. The mutation type corresponding to the maximum probability (confidence score) is taken as the predicted type. If there is a tie for the maximum probability, the tie is broken by the order LoF, SoF, CoF, GoF. For a given variant *v*_*i*_ and predefined cutoff *t*, ${S_{i}^{0}}>t$ indicates that in the target “subfamily”, the wild type sequence fits better than the mutant type sequence, so there is a higher probability of losing the original function. Further, if for the “subfamilies” *x*, from which the minimum HMMvar score is calculated, the wild type sequence fits better than the mutant type sequence, then no new function is acquired and results in LoF (${L_{i}^{0}}>0.5$ and ${A_{i}^{x}}<0.5$). Otherwise, *v*_*i*_ is classified as SoF (${L_{i}^{0}}>0.5$ and ${A_{i}^{x}}\ge 0.5$) with higher confidence score, because although *v*_*i*_ probably causes the protein loss of function in subfamily *C*_0_, *v*_*i*_ obtains the specific function in some *C*_*m*_. On the other hand, if ${S_{i}^{0}} \le t$, the variant could potentially cause gain of function. Then if the mutant type sequence fits better in subfamily *x* (${S_{i}^{x}}<t$), which means there exists at least one other “subfamily” that the mutant type sequence fits better than the wild type sequence, the variant *v*_*i*_ is classified as GoF (${L_{i}^{0}} \le 0.5$ and ${A_{i}^{x}}>0.5$) with higher confidence score; otherwise, *v*_*i*_ is classified with CoF (${L_{i}^{0}} \le 0.5$ and ${A_{i}^{x}}\le 0.5$).

## Results

### Prediction of TSHR mutations

**Fig. 4:**
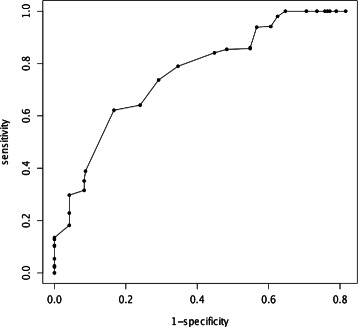
Receiver operating curve (ROC) for prediction of TSHR mutations. Receiver operating curve (ROC) for prediction of TSHR mutations (sensitivity is with respect to GoF; the area under curve (AUC) is 0.613)

Thyroid-stimulating hormone (TSH, thyrotropin) and its receptor TSHR together play a key role in controlling thyroid function. Mutations in TSHR can be loss of function or gain of function, leading to hypo or hyperthyroidism, respectively. The discovery of large serial gain of function mutations in TSHR is of great interest, revealing a new disease mechanism of mutations that constantly increase the basal activity of a receptor [21]. 111 TSHR mutations were collected from the TSHR Mutation Database and their functional outcomes predicted. Table 1 shows the result. Prediction is not available for three of the variants because the bit scores calculated are not significant. For the remaining 108 mutations, 61 are annotated by the database as “gain of function”, 47 “loss of function”. HMMvar-func predicts 39 gain of function (GoF) mutations, 25 loss of function (LoF), 42 switch of function (SoF), and two conservation of function (CoF). As only two types of mutations, LoF and GoF, are annotated by the database, the predicted 25 LoF and 39 GoF mutations are used to calculate the performance metrics. Figure 4 shows the ROC with respect to *t* for HMMvar-func based on CEO clustering. The best performance is achieved at *t*=2.7 with sensitivity 78.9 % (with respect to GoF), specificity 65.4 %, and accuracy 73.4 %. The predicted types with high confidence scores are more reliable, thus it is reasonable to focus on these variants, which also avoids the ambiguity of confidence score ties. Considering only the variants with the maximum confidence score greater than 0.5 (33 in total, 18 GoF and 15 LoF), the sensitivity (with respect to GoF), specificity, and accuracy are 85.7 %, 68.2 %, and 76.7 %, respectively. The detailed confidence scores are in Additional file 1: Table S1. The CEO algorithm automatically determines the number of clusters to minimize (locally) the combinatorial entropy [20]. Due to the processing of the MSA, the MSA used for the clustering step is a segment of the original MSA, and this segment is possibly different for different variants. As a result, the number of clusters generated by the CEO algorithm is not fixed for all the variants. The average number of clusters generated in this data set is 19 from 162 sequences in the original MSA (excluding the clusters with size one).

**Table 1:** The confusion matrix of the prediction results for the TSHR mutations. The rows correspond to the database annotation, the columns the predicted categories

	GoF	LoF	SoF	CoF
GoF	30	8	23	0
LoF	9	17	19	2

**Fig. 5:**
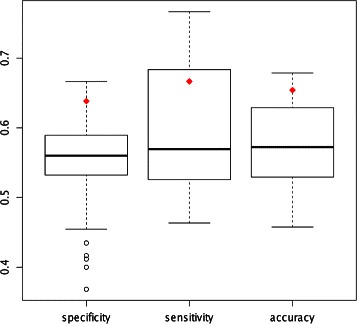
The performance of HMMvar-func based on K-means clustering or CEO clustering. (100 random initial guesses are evaluated for K-means clustering on the TSHR data set with k = 4 and t = 2:7. The red diamond points represent the corresponding performance of the CEO clustering)

Two aspects of the HMMvar-func prediction method merit investigation, the clustering method and the cutoff score *t* set in Fig. 4. The present work uses the CEO algorithm suggested in [20]. The *K*-means clustering method, used in previous work [22], is compared with the CEO algorithm in Fig. 5 (*k*=4). The *K*-means clustering is extremely sensitive to the initial guesses, so 100 runs with random initial guesses are performed to reduce this effect. The number of clusters generated by the CEO method is controlled to be the same as in the *K*-means clustering (*k*=4) for a fair comparison. Figure 5 shows that the CEO statistics are much better than what would be expected from using *K*-means, but that the CEO clusters are not optimal, and a lucky *K*-means clustering can do much better than CEO.

The inner coherence of the clusters generated by CEO and *K*-means is also compared in Table 2. The “median” and “best” *K*-means are defined in terms of the median and best accuracy shown in Fig. 5, respectively. The Dunn index and Davies-Bouldin index are consistent with the accuracy metrics. Better cluster quality corresponds to a higher Dunn index and a lower Davies-Bouldin index.

**Table 2:** The comparison of CEO and *K*-means

	Dunn	Davies-Bouldin	Accuracy	Sensitivity	Specificity
CEO	0.429	0.838	0.654	0.667	0.638
median *K*-means	0.378	0.973	0.574	0.569	0.560
best *K*-means	0.513	0.839	0.679	0.742	0.600

As expected, results here demonstrate that both the clustering method and the cutoff score *t* can affect the prediction results, the better the cluster quality, the more accurate the prediction. Since there is no consensus on which clustering method works best, and clustering algorithms can find only a locally optimal clustering, it is advisable to perform multiple clusterings, and use only the best (by Dunn index, e.g.) clusters for downstream prediction.

### Switch of function

**Fig. 6:**
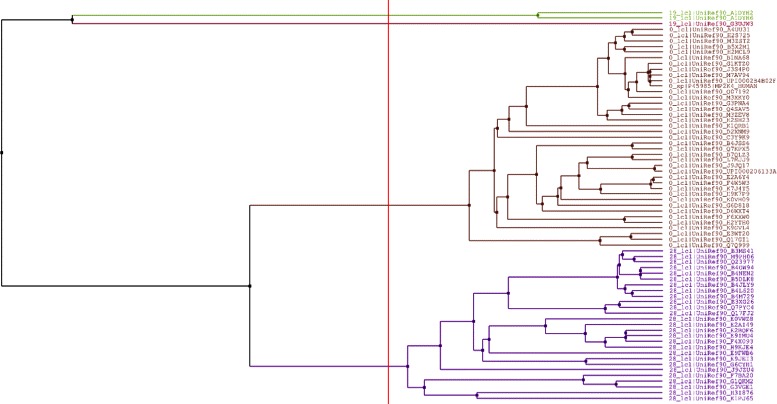
Distance tree of the MAP2K subfamilies. Colors indicate different subfamilies. The minimum score ${S_{i}^{x}}$ is calculated from *C*_19_. *C*_0_ is the target cluster. *C*_28_ is an example subfamily that the mutant protein could switch to. The leaves are protein sequences. Two sequences are merged according to the BLOSUM62 matrix by averaging the substitution distance over all the positions in the MSA. The numeric prefix of a sequence ID is the cluster number

The switch of function mutations reported in [8] are tested. The R132H mutation in IDH1, shown experimentally [23] to lead to loss of the original function but gain of new function, essentially falls into the category of “switch of function” defined in the current study, and is also investigated here. As shown in Table 3, three mutations (in PTPRD, MAP2K4, CDH1) are predicted as switch of function with confidence score over 0.6. As an example, Fig. 6 shows the tree generated by Jalview [24] from the processed alignment of homologous sequences of the MAP2K4 protein (trees for RAC1, PTPRD, and CDH1 are shown in Additional file 2: Figures S1–S3). The tree is built according to the average distance using BLOSUM62 and based on sum of scores for the residue pairs at each aligned position. The tree shows three clusters, *C*_19_, *C*_28_, and *C*_0_, with *C*_0_ being the target cluster. The minimum score ${S_{i}^{x}}$ is calculated from *C*_19_.

**Table 3:** Switch of function mutations

Gene	Variant	Predicted type	Confidence score
RAC1	A95E	SoF	0.548
PTPRD	R28Q	SoF	0.728
MAP2K4	Q142L	SoF	0.800
CDH1	H233Q	SoF	0.651
IDH1	R132H	GoF	0.533

According to the HMMvar scores, *C*_19_ and *C*_28_ are the potential subfamilies that the protein MAP2K4 might switch to due to Q142L (not all the potential subfamilies are listed). Q142L, a missense mutation, has been identified as one of the major somatic mutations in human lung cancer samples [25]. It is predicted to be “damaging” by SIFT [1]. However, another commonly used programs PolyPhen-2 [2] predicts it as “neutral”. The HMMvar-func prediction together with [8] suggests an alternative hypothesis for the functional impact of the variant, namely “switch of function” in MAP2K4, which seems to be more likely considering its common occurrence in lung cancer samples [25].

Similarly, the two mutations in PTPRD and CDH1 are likely to lead to switch of function with high probability. PTPRD has been found to be somatically mutated in colorectal carcinoma with the R28Q mutation [26]. H233Q in CDH1 was found to be associated with breast cancer [27].

The prediction for A95E in RAC1 gene is switch of function. However, the confidence score is only slightly greater than 0.5, because the probability ${L_{i}^{0}}$ (Fig. 3) of losing the original functions is low (0.55) whereas the probability ${A_{i}^{x}}$ of acquiring new functions is high (0.997), making a switch of function classification unreliable. Previous studies are more agreed on the ‘gain of function’ prediction. As discussed before, the cutoff *t* is an important factor in determining the final prediction. If *t*=3.0 instead of 2.7, A95E is predicted as gain of function with confidence score 0.524. Similarly the R132H mutation in IDH1 is predicted as gain of function with low confidence score (${L_{i}^{0}}=0.40$, ${A_{i}^{x}}=0.89$). The confidence score calculation assumes the independence of losing the original functions and gaining new functions. As a result, for those variants with low confidence scores, the probability of losing the original functions (${L_{i}^{0}}$) and the probability of acquiring new functions (${A_{i}^{x}}$) should both be considered.

### Application to cancer mutations

**Fig. 7:**
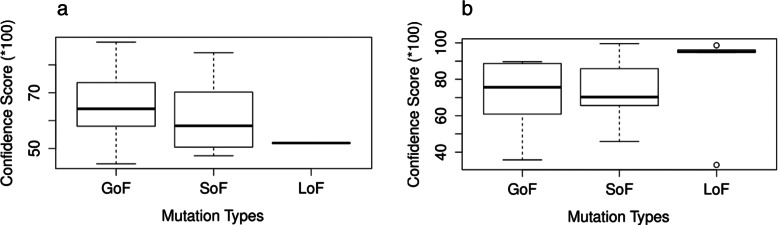
Confidence score distribution for different predicted mutation types: **a** confidence score for EGFR mutations, **b** confidence score for BRAF mutations

Oncogenic mutations in the EGFR gene and the BRAF gene [16] are evaluated. All the variant data are listed in Additional file 1: Table S3. Activating mutations in EGFR and BRAF are frequently found to be associated with cancer [28–31]. Improper activation results in increased malignant cell survival, proliferation, invasion, and metastasis. Table 4 shows the total number of activating (GoF) mutations evaluated and the corresponding number of predicted GoF, SoF, LoF, and CoF classifications for each gene. The predicted types are dominated by gain of function and switch of function classifications as expected, because the GoF and SoF mutations are both expected to have the protein acquiring new functions. The median confidence score for GoF is greater than that for SoF, which means the mutant gene is more likely to keep the original functions. Distribution details of the confidence scores for both genes are in Fig. 7.

**Table 4:** Prediction of oncogenic mutations

Gene	Total	GoF	SoF	LoF	CoF
EGFR	78	31	44	1	0
BRAF	46	13	27	5	0

**Fig. 8:**
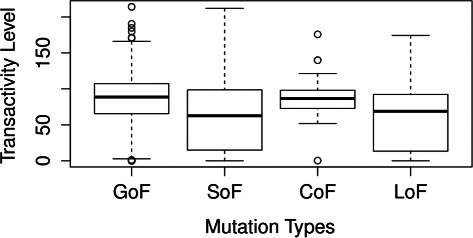
The transactivity level of the gene TP53 in different predicted mutation groups

The predicted types for TP53 mutations are compared against the transactivity level as shown in Fig. 8. The medians of the transactivity level in the GoF and CoF groups are higher than those in the SoF and LoF groups, as ‘loss of function’ mutations inactivate tumor suppressor genes and the genes are likely losing the original functions as a result of LoF or SoF. The LoF variants predicted by HMMvar-func were also scored by HMMvar, and results show that a majority of them (70 %) have scores greater than 2, which is considered by HMMvar to be deleterious.

In [32], the authors concluded that the mutants of TP53 on the 273rd codon show growth modulation activities regardless of its specific transactivation. Specifically, the R273H mutation enhances cell growth in spite of its reservation of transactivation activity, whereas the R273L mutation suppresses cell growth in spite of its complete loss of the TP53 specific transactivation. HMMvar-func predicts R273H to be gain of function mutation and R237L switch of function mutation. Therefore, the HMMvar-func prediction of the functional outcome of these two mutations is indeed consistent with the finding in [32].

## Discussion

This paper, based on previous work [14], proposes using multiple hidden Markov models to predict the fine grained functional impact of mutations on proteins. A soft classification of functional outcome type based on the logistic function and combinatorial probabilities follows HMMvar scoring. The prediction pipeline is applied to various datasets with positive results, providing evidence that the pipeline is capable of identifying different types of mutations.

This paper is the first to computationally define functional impact of mutations: loss, switch, gain, and conservation of function. Sequences homologous to the gene with mutations are clustered as protein families or subfamilies, which are represented by profile HMMs that implicitly capture evolutionary/functional information. Thus computing the fitness of a sequence against the profiles indicates the functional transfer among subfamilies. The HMMs, rather than focusing on a specific position or the mutant position as some evolutionary analysis methods do, consider a region extended from the mutant position.

The quality of the MSA is important to the prediction performance. The MSA processing step in the pipeline keeps the homologous sequences and removes redundant sequences over an alignment similarity threshold; low quality columns are also eliminated. Finally, the proper region is selected by left and right extension from the position of the variant. The cluster quality also affects the prediction. Rather than tinkering with some variant of *K*-means clustering to find the correct number of clusters and avoid local optimal solutions, the CEO [20] algorithm is used in the prediction pipeline. The CEO algorithm achieves good clustering (also possibly only locally optimal) by considering conservation in both the overall family and subfamilies.

Note that the traditional definition of GoF [20], includes both those variants that acquire a new function while maintaining the original one and also the ones that enhance the original function. The GoF defined in this paper is limited to only the former case.

Prediction of the functional impact of variants, such as deleterious or neutral, is important, but computationally predicting the fine grained type of mutations is equally crucial, especially in cancer studies. These fine grained predictions can be used to target mutated genes and mutations that play crucial roles in resistance to certain therapeutic agents.

## Conclusion

This work presents HMMvar-func, a new method for predicting the functional outcome of mutations in coding regions. The fine grained prediction provides richer information than current existing tools that can be especially useful for studying mutations in cancer. The prediction can be used to help filtering and identifying from many coding variants the ones that truly contribute to the disease/cancer of interest, thus serving as a prioritization tool for variants for further downstream studies.

## Additional files

Additional file 1
**Supplementary Tables.** (XLSX 323 kb)

Additional file 2
**Supplementary Figures.** (PDF 102 kb)
