# Supplementary material for: HMMvar-func: a new method for predicting the functional outcome of genetic variants
Source: BMC Bioinformatics. 2015 Oct 30;16:351. doi: 10.1186/s12859-015-0781-z (PMC4628267; doi:10.1186/s12859-015-0781-z)
Supplement: Additional file 2 — Supplementary Figures. (PDF 102 kb) [file 12859_2015_781_MOESM2_ESM.pdf]

HMMvar-func: a new method for predicting the functional outcome of genetic variants

Mingming Liu, Layne T. Watson, Liqing Zhang

Supplement 2: Distance tree of subfamilies

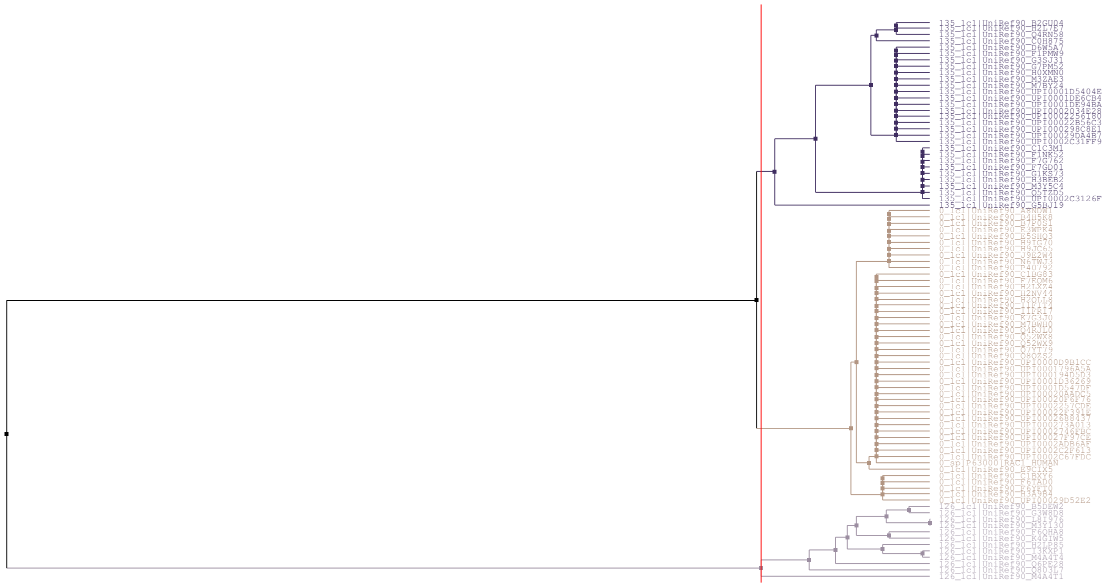

Figure 1: Distance tree of the RAC1 subfamilies (n = 836, k = 140). Colors indicate different subfamilies. The minimum score S_i^x is calculated from C_{126}. C_0 is the target cluster. C_{135} is an example subfamily that the mutant protein could switch to. The leaves are protein sequences. Two sequences are merged according to the BLOSUM62 matrix by averaging the substitution distance over all the positions in the MSA.

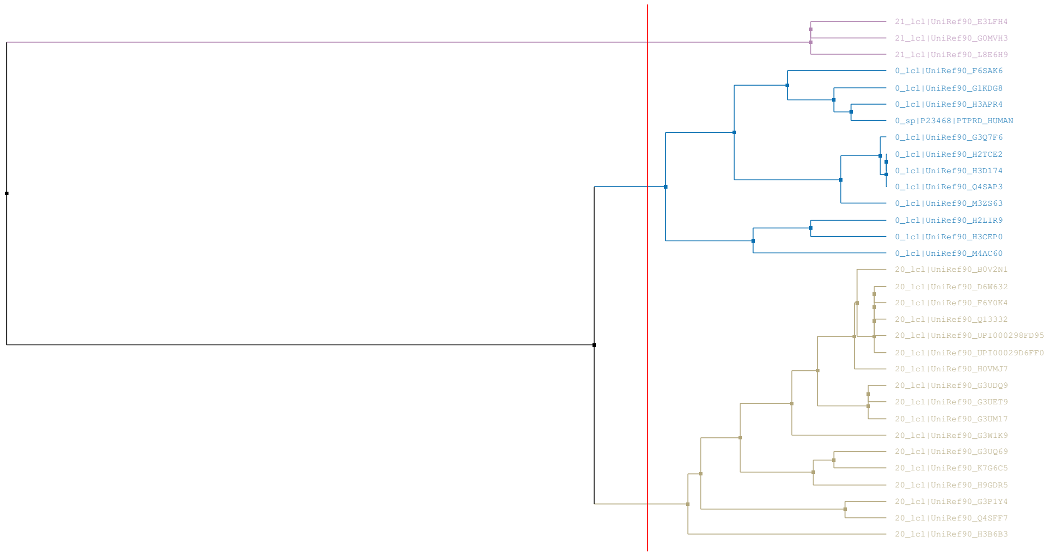

Figure 2: Distance tree of the PTPRD subfamilies ($n = 75$, $k = 21$). Colors indicate different subfamilies. The minimum score S_i^x is calculated from C_{21}. C_0 is the target cluster. C_{20} is an example subfamily that the mutant protein could switch to. The leaves are protein sequences. Two sequences are merged according to the BLOSUM62 matrix by averaging the substitution distance over all the positions in the MSA.

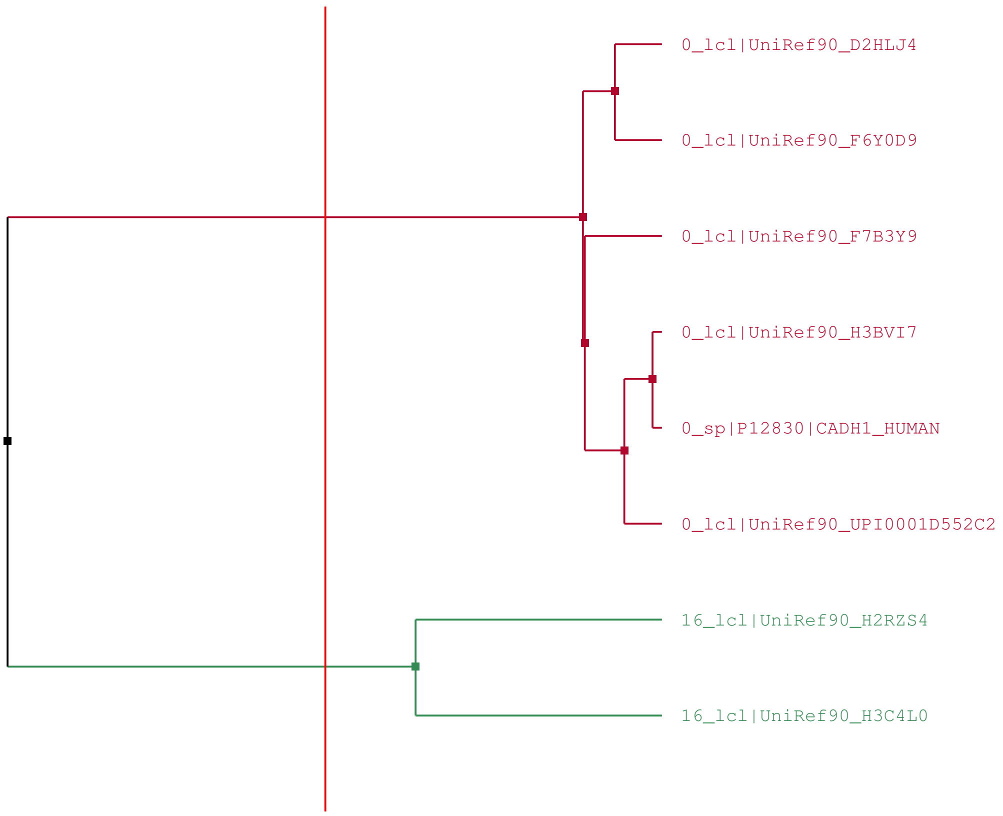

Figure 3: Distance tree of the CDH1 subfamilies ($n = 76$, $k = 29$). Colors indicate different subfamilies. The minimum score S_i^x is calculated from C_{16}. C_0 is the target cluster. The leaves are protein sequences. Two sequences are merged according to the BLOSUM62 matrix by averaging the substitution distance over all the positions in the MSA.

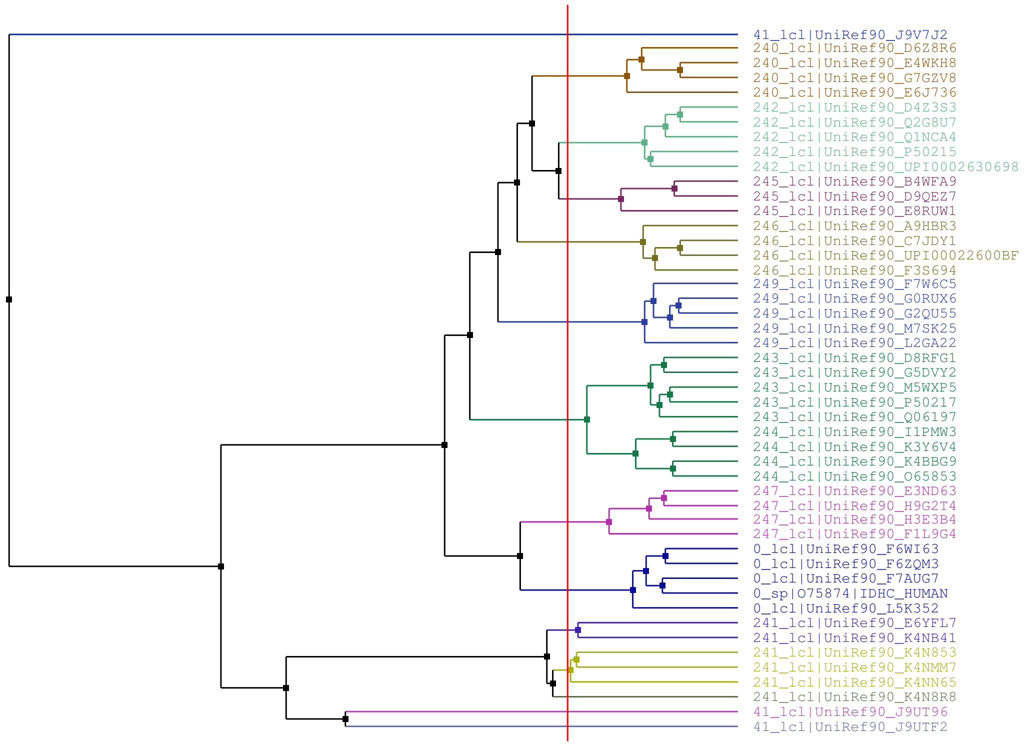

Figure 4: Distance tree of the CDH1 subfamilies ($n = 826$, $k = 274$). Colors indicate different subfamilies. The minimum score S_i^x is calculated from C_{41}. C_0 is the target cluster. The leaves are protein sequences. Two sequences are merged according to the BLOSUM62 matrix by averaging the substitution distance over all the positions in the MSA.
